# Gut microbiota regulates mouse behaviors through glucocorticoid receptor pathway genes in the hippocampus

**DOI:** 10.1038/s41398-018-0240-5

**Published:** 2018-09-07

**Authors:** Yuanyuan Luo, Benhua Zeng, Li Zeng, Xiangyu Du, Bo Li, Ran Huo, Lanxiang Liu, Haiyang Wang, Meixue Dong, Junxi Pan, Peng Zheng, Chanjuan Zhou, Hong Wei, Peng Xie

**Affiliations:** 1 Institute of Neuroscience and the Collaborative Innovation Center for Brain Science, Chongqing Medical University, Chongqing, 400016 China; 2 Chongqing Key Laboratory of Neurobiology, Chongqing, 400016 China; 3 Department of Neurology, Yongchuan Hospital, Chongqing Medical University, Chongqing, 402160 China; 4 Department of Laboratory Animal Science, College of Basic Medical Sciences, Third Military Medical University, Chongqing, 400038 China; 5 Department of Nephrology, the Second Affiliated Hospital of Chongqing Medical University, Chongqing, 400010 China; 6 Key Laboratory of Clinical Laboratory Diagnostics (Ministry of Education), Chongqing, China; 7 Department of Neurology, the First Affiliated Hospital of Chongqing Medical University, Chongqing, 400042 China; 8 South Australian Health and Medical Research Institute, Mind and Brain Theme, and Flinders University, Adelaide, SA Australia

## Abstract

Gut microbiota has an important role in the immune system, metabolism, and digestion, and has a significant effect on the nervous system. Recent studies have revealed that abnormal gut microbiota induces abnormal behaviors, which may be associated with the hypothalamic–pituitary–adrenal (HPA) axis. Therefore, we investigated the behavioral changes in germ-free (GF) mice by behavioral tests, quantified the basal serum cortisol levels, and examined glucocorticoid receptor pathway genes in hippocampus using microarray analysis followed by real-time PCR validation, to explore the molecular mechanisms by which the gut microbiota influences the host’s behaviors and brain function. Moreover, we quantified the basal serum cortisol levels and validated the differential genes in an *Escherichia coli-*derived lipopolysaccharide (LPS) treatment mouse model and fecal “depression microbiota” transplantation mouse model by real-time PCR. We found that GF mice showed antianxiety- and antidepressant-like behaviors, whereas *E. coli* LPS-treated mice showed antidepressant-like behavior, but did not show antianxiety-like behavior. However, “depression microbiota” recipient mice exhibited anxiety- and depressive-like behaviors. In addition, six glucocorticoid receptor pathway genes (*Slc22a5*, *Aqp1*, *Stat5a*, *Ampd3*, *Plekhf1*, and *Cyb561*) were upregulated in GF mice, and of these only two (*Stat5a* and *Ampd3*) were upregulated in LPS-treated mice, whereas the shared gene, *Stat5a*, was downregulated in “depression microbiota” recipient mice. Furthermore, basal serum cortisol levels were decreased in *E. coli* LPS-treated mice but not in GF mice and “depression microbiota” recipient mice. These results indicated that the gut microbiota may lead to behavioral abnormalities in mice through the downstream pathway of the glucocorticoid receptor. Herein, we proposed a new insight into the molecular mechanisms by which gut microbiota influence depressive-like behavior.

## Introduction

The human gastrointestinal tract contains approximately trillion microorganisms, most of which are bacteria, belonging to > 1000 bacterial species^1^. Bacteroidetes and Firmicutes constitute over 90% of the species in the gut microbiota^2^. Preclinical and clinical evidence have indicated that the gut microbiota plays an important role in a wide range of host processes in both healthy population and patients^3,4^. In particular, recent studies have hinted that the gut microbiota can have dramatic effects on the development and function of the host brain^5^.

Recently, accumulating studies have revealed bidirectional communication between the intestinal microbiota and the brain, and proposed a novel conceptual model of a “microbiota–gut–brain axis”^6–8^. The gut–brain axis is an interaction system that integrates immunological, neural, endocrine and metabolic pathways, and dysfunction of this axis has pathophysiological effects on the host^9,10^. Many studies have found a high prevalence of mood disorders such as depression and/or anxiety in patients with gastrointestinal disorders including irritable bowel disorder and inflammatory bowel disorder^11,12^, which indicated the importance of the gut–brain axis in the pathophysiology of mood disorders. The gut and the brain may communicate through multiple mechanisms, including immune responses, vagus nerve, short–chain fatty acids, endocrine signaling and tryptophan metabolism^5,13^. Our previous studies have revealed that the gut microbiota can modulate anxiety- and depressive-like behaviors in mice^14–16^, and lead to molecular changes in the hippocampus^17,18^, hypothalamus^15^, and liver^16^. However, the molecular mechanisms of the communication between gut microbiota and brain are not fully understood.

Germ-free (GF) mice, born and raised without exposure to microbes, is a powerful tool for understanding the effects of the gut microbiota on the host. Previous studies have demonstrated that GF mice had higher levels of serum adrenocorticotrophic hormone (ACTH) and corticosterone following acute stress compared with specific pathogen-free (SPF) mice^19,20^. Moreover, GF mice exhibited antianxiety-like behavior and increased plasma corticosterone levels compared with SPF mice, and the elevated plasma corticosterone may also be owing to the stress of acclimatization^21^. GF rats showed increased peripheral corticosterone levels after stress and a decreased glucocorticoid receptor (*Nr3c1*) mRNA level in the hippocampus^22^. In addition, plasma ACTH and corticosterone levels were decreased in *Bifidobacterium infantis*-monoassociated mice, whereas they were increased in *Escherichia coli–*monoassociated mice^20,23^, suggesting the important role of corticosterone in gut microbiota alterations. Therefore, we speculated that the disturbances in glucocorticoid receptor pathway are the important mechanisms by which the gut microbiota influences the host’s behaviors and brain function.

To test this hypothesis, we investigated the behavioral changes in GF mice, the basal serum cortisol levels and the alterations in expression of glucocorticoid receptor pathway genes in hippocampus. More importantly, we compared the present results with those of the *E. coli-*derived lipopolysaccharide (LPS) treatment mouse model and the fecal “depression microbiota” transplantation (FMT) mouse model. Finally, we further analyzed the expression alterations of the glucocorticoid receptor pathway genes in hippocampus accompanied with behavioral changes, to explore the potential mechanisms by which the gut microbiota influences the host’s behaviors.

## Material and methods

### Animals

Eight-week-old male GF (*n* = 19) and SPF (*n* = 20) BALB/c mice were provided by the animal experimental center of the Third Military Medical University (Chongqing, China). GF mice were fed in flexible film plastic isolators. All conditions were kept sterile and verified to meet the Chinese Laboratory Animal Microbiological Standards and Monitoring (GB 14922.2-2011) by testing the feces and skin of the GF mice. GF and SPF mice were housed five per cage under a 12-h light/dark cycle (lights on at 07:30–19:30), a constant temperature of 21–22 °C and a relative humidity of 55 ± 5%. Autoclaved standard mice chow of the same formulation and water ad libitum was given to all animals. This study was approved by the Ethics Committee of Chongqing Medical University and conducted in accordance with the National Institutes of Health Guide for the Care and Use of Laboratory Animals (NIH Publication No. 80-23).

### *E. coli* LPS-treated mice

Thirteen 6-week-old male SPF mice were given daily drinking water containing *E. coli* LPS (Sigma-Aldrich Ltd., St. Louis, MO, USA) at a concentration of 20 μg/ml for 2 weeks, and the consumption of drinking water was not limited. The control SPF mice (male, 6 weeks old, *n* = 13) were given daily drinking water without *E. coli* LPS. All other conditions between the two groups were consistent. Behavioral tests were performed two weeks later, followed by tissue sample collection.

### FMT mice

As previously described^14^, the FMT mouse model (*n* = 30) was established by transferring fecal samples of severe depressive patients to GF mice, and the control mice (*n* = 30) were transplanted with normal human feces. The two groups were called “depression microbiota” recipient mice group and “healthy microbiota” recipient mice group, respectively. Behavioral tests were performed, and tissue samples were collected after 2 weeks. In addition, the timelines of above-mentioned mouse models were displayed in Supplementary method. S1.

### Open field test (OFT)

The GF and SPF mice were moved to the testing room for acclimation at least 1 h prior to behavioral testing. Mice were placed individually in the middle of the open field apparatus (45 × 45 × 45 cm) and allowed to adapt for 1 min, then the spontaneous activities were recorded for 5 min using the SMART 2.5 software (Panlab, Barcelona, Spain). The total movement distance and the percentage of center distance (inner 25% of the surface area) were used as indexes of locomotor activity and anxiety-like behavior, respectively. After each mouse was tested, the odor was eliminated with 70% alcohol.

### Forced swim test (FST)

Mice were placed in a Plexiglas cylinder (30 cm high, 15 cm diameter) filled with water (25 ± 1°C) to 18 cm. The test lasted 6 min, and in the last 5 min we recorded the immobility/absence of all motion with the exception of minimal movements to keep the head above the water. Immobility time was used to evaluate the depressive-like behavior of the mice. The water was completely replaced after each test.

### Novelty suppressed feeding test (NSFT)

All mice were food-deprived for 24 h prior to the test. Each mouse was placed in the corner of the open field apparatus (45 × 45 × 45 cm) with a single food pellet in the center. The latency to feed, an index of depressive-like behavior and motivation level of the mouse, was recorded. Then, the mouse was immediately returned to its home cage and the food consumption during the subsequent 5 min was measured to eliminate the effect of appetite on the latency time.

### Sample collection and preparation

Mice were killed in a random order by cervical dislocation after being anesthetized with 10% chloral hydrate (400 mg/kg)^15^. The brain was quickly removed from the cranium, and hippocampus were dissected out on ice-cold plate and frozen in liquid nitrogen, then stored at −80 °C before assay. Blood samples were collected in a plastic tube. The serum was obtained by centrifugation (3000 *g*, 10 min, 4 °C) and stored at −80 °C for subsequent analysis. To avoid fluctuations in the results owing to the circadian rhythm of hypothalamic–pituitary–adrenal (HPA) axis, samples in each group were collected at the same time of day (between 9:00 and 11:00 h)^20^.

### Microarray analysis

The Glucocorticoid Signaling PCR Microarrays (SABiosciences Corporation, Frederick, MD, USA) were used to analyze the alterations in glucocorticoid receptor pathway genes. In detail, three arrays were used for the GF mice and three for the SPF mice. Three mouse hippocampus were mixed into a pooled RNA sample, and all experiments in GF mice group and SPF mice group were performed in triplicate using distinct RNA samples. Total RNA from each sample were extracted with TRIzol extraction procedures (Invitrogen, Carlsbad, CA, USA). Then, the total RNA from each sample were purified by RNeasy® MinElute™ Cleanup Kit (Invitrogen) and the RNA concentration and purity were tested by NanoDrop ND-1000 spectrophotometer (Thermo Fisher Scientific, Waltham, MA, USA). The RNA samples were examined for the 84 related genes in the glucocorticoid receptor pathway (Supplementary Table. 1). Data were analyzed with the 2^-ΔΔCt^ method and exhibited in the form of fold change. Student’s *t* test was used to select the different genes between the two groups (*p* < 0.05). The *q* value method was employed to control the false discovery rate (set to 0.1) associated with multiple analysis^24,25^.

### Bioinformatics analysis

Ingenuity Pathway Analysis (IPA) software (Qiagen, Shanghai, China) was used to analyze the biological functions and relevant networks of differentially expressed genes, and the enrichment of genes and diseases/functions.

### Quantitative real-time PCR (qRT-PCR)

qRT-PCR was conducted as previously described^18^. In brief, RNA was extracted from hippocampal tissues of mice according to the TRIzol extraction protocol (Thermo Fisher Scientific, MA, USA), and reverse-transcribed into cDNA with the PrimeScript™ RT reagent Kit (Takara, Dalian, China). Subsequently, qRT-PCR was performed with the LightCycler 96 system (Roche, Mannheim, Germany) with GoTaq® 1-Step RT-qPCR System for Dye-Based Detection (Promega, Madison, WI, USA). The primers were obtained from Sangon Biotech (Shanghai, China). Geometric mean of *Gapdh* and *Actb* mRNA was used to normalize the target mRNA^26^.

### Serum cortisol measurement

The basal serum cortisol levels of three mice models were quantified using a Mouse Cortisol Enzyme-linked Immunosorbent Assay (ELISA) Kit (Sangon Biotech, Shanghai, China) as recommended by the manufacturer’s protocol. Optical absorbance at 450 nm was measured with a microplate reader (Bio-Rad Co., California, USA). A standard curve was constructed (15–240 ng/ml) and the concentrations of cortisol in serum samples were determined from the standard curve.

### Statistical analysis

The results were analyzed using nonparametric tests or Student’s *t* test, where appropriate. All statistical analyzes were performed using SPSS 20.0 software (IBM, Armonk, NY, USA). Data are presented as mean ± SEM. A value of *p* < 0.05 was considered statistically significant.

## Results

### GF mice exhibit antianxiety- and antidepressant-like behaviors

**Fig. 1:**
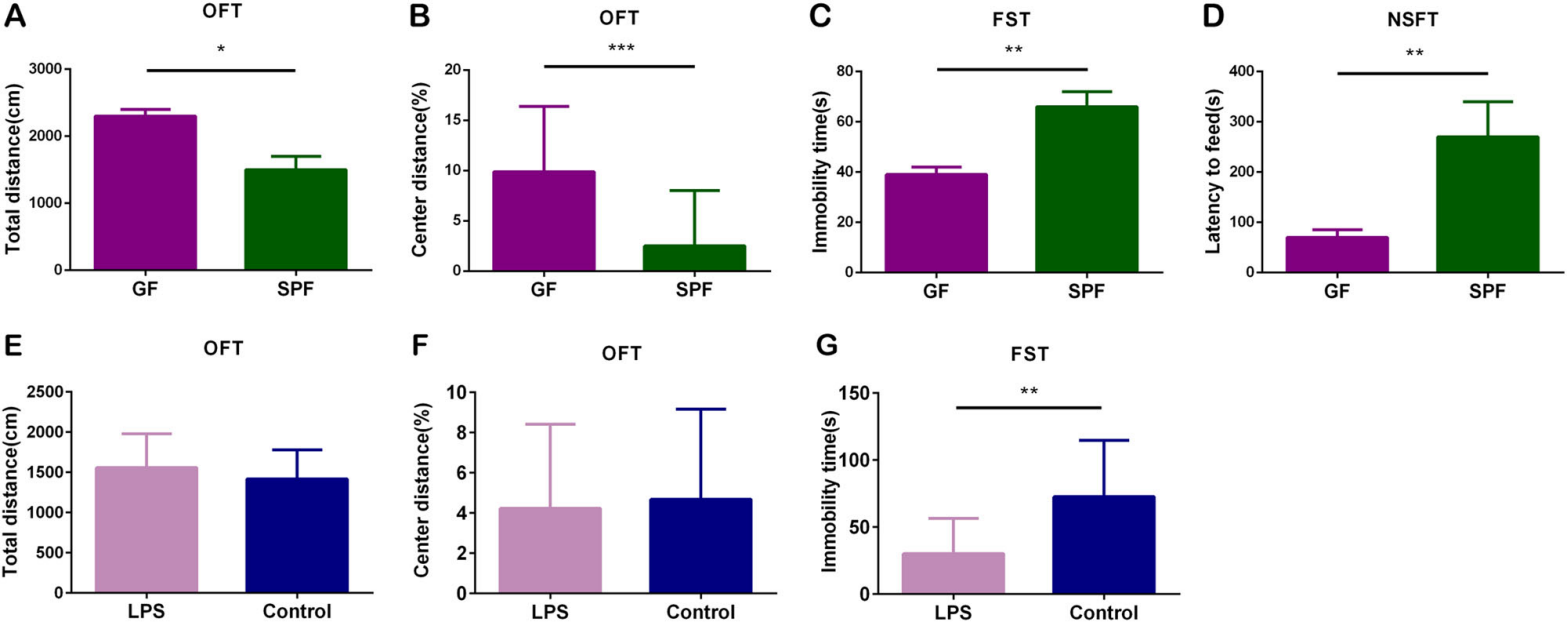
Absence of microbiota leads to antianxiety- and antidepressant-like phenotypes, and *E. coli* lipopolysaccharide (LPS) treatment leads to an antidepressant-like phenotype. **a**–**d** The total distance **a** and the percentage of center distance **b** in the open field test (OFT), the immobility time **c** in the forced swim test (FST), and the latency to feed **d** in the novelty suppressed feeding test (NSFT) of germ-free (GF) (*n* = 19) and specific pathogen-free (SPF) (*n* = 20) mice. **e**–**g** The total distance **e** and the percentage of center distance **f** in the OFT, and the immobility time **g** in the FST of *E. coli* LPS-treated mice (*n* = 13) and control mice (*n* = 13). Data represent the mean ± SEM. * *p* < 0.05, ** *p* < 0.01, *** *p* < 0.001

As previously described, we explored the effects of gut microbiota on mice behaviors by comparing GF and SPF mice. In the OFT, the GF mice showed a significantly increased total distance (Fig. 1a, *p* < 0.05) and increased percentage of center distance (Fig. 1b, *p* < 0.001) compared with the SPF mice, indicating higher locomotor activity and antianxiety-like behavior in GF mice. Moreover, the GF mice exhibited less immobility time than the SPF mice in the FST (Fig. 1c, *p* < 0.01) and showed decreased latency to feed in the NSFT (Fig. 1d, *p* < 0.01), suggesting antidepressant-like behavior in GF mice.

### *E. coli* LPS-treated mice show antidepressant-like behavior

In current study, *E. coli* LPS-treated mice showed less immobility time than the control mice in the FST (Fig. 1g, *p* < 0.01). However, in OFT, there were no differences in the total distance (Fig. 1e) or the percentage of center distance (Fig. 1f) between *E. coli* LPS-treated mice and control mice. These results suggest that the *E. coli* LPS-treated mice exhibit antidepressant-like behavior, but not antianxiety-like behavior.

### “Depression microbiota” recipient mice exhibit anxiety- and depressive-like behaviors

Previously we have reported the “depression microbiota” recipient mice exhibited anxiety- and depressive-like behaviors compared with “healthy microbiota” recipient mice^14^. Namely, in OFT, the percentage of center distance of “depression microbiota” recipient mice was decreased compared with “healthy microbiota” recipient mice. Moreover, the immobility time in FST and tail suspension test was increased in “depression microbiota” recipient mice.

### Absence of gut microbiota upregulates the expression of glucocorticoid receptor pathway genes

We examined 84 genes related to the glucocorticoid receptor pathway in the hippocampus of GF and SPF mice by using microarray. A total of 23 genes showed significant differences between the two groups (*p* value < 0.05, FDR < 0.1) (Table 1). Among them, *Slc10a6* (fold change (FC) = 22.81), *Ghrhr* (FC = 11.99), and *Aqp1* (FC = 7.39) had the highest FC values. These results revealed that the absence of gut microbiota significantly upregulated the glucocorticoid receptor pathway genes in hippocampus.

**Table 1:** Differentially expressed genes in the microarray

Gene	FC	*p* value	FDR	Gene	FC	*p* value	FDR
Slc10a6	22.81	< 0.001	< 0.001	Pou2f1	1.29	0.021	0.042
Slc22a5	1.46	0.002	0.025	Bmper	1.33	0.023	0.042
Tsc22d3	2.30	0.004	0.025	Bcl6	1.28	0.023	0.042
Aqp1	7.39	0.005	0.025	Plekhf1	2.06	0.028	0.043
Mertk	1.73	0.005	0.025	Creb1	1.21	0.028	0.043
Stat5a	1.55	0.006	0.028	Arid5b	1.33	0.029	0.043
Ampd3	1.40	0.008	0.029	Adarb1	1.18	0.031	0.043
Ghrhr	11.99	0.009	0.030	Sgk1	1.93	0.032	0.043
Pld1	1.55	0.011	0.034	Cyb561	1.24	0.034	0.044
Fkbp5	1.32	0.013	0.036	Usp2	1.17	0.044	0.054
Aff1	1.38	0.020	0.042	Glul	1.18	0.049	0.058
Per1	1.26	0.020	0.042				

### Differentially expressed genes in hippocampus are associated with neurological disorders

We used IPA software to analyze the 23 differentially expressed genes and found that these genes were significantly enriched in neurological disorders (Supplementary Figure S1) and highly related to cell development, cell proliferation, cell death, and cell survival (Supplementary Figure S2).

### qRT-PCR validation

**Fig. 2:**
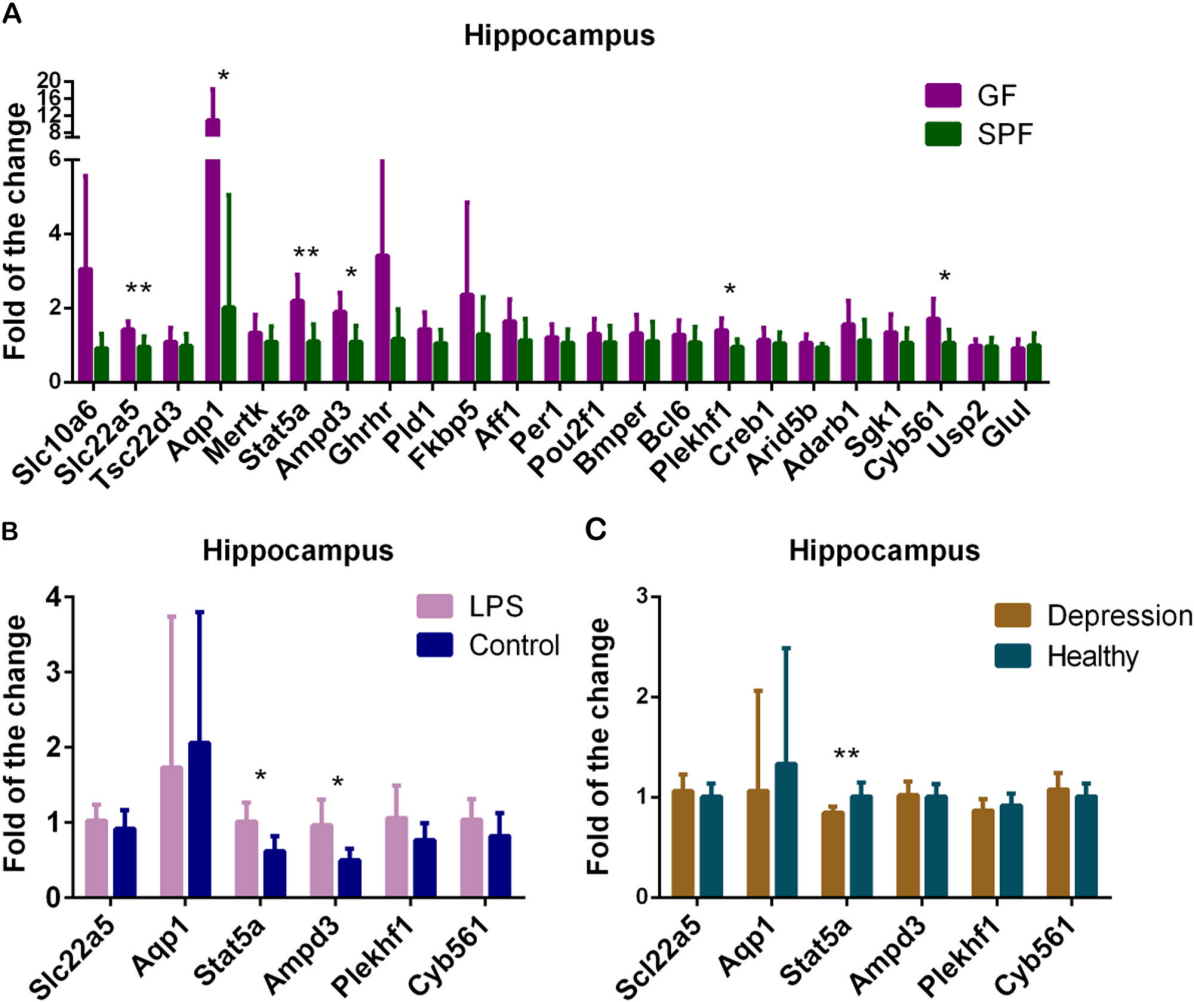
Real-time PCR validation of the expression of glucocorticoid receptor pathway genes in three mouse models. **a** Real-time PCR validation of the 23 differentially expressed glucocorticoid receptor pathway genes detected by the microarray analysis between germ-free (GF) mice (*n* = 8) and specific pathogen-free (SPF) mice (*n* = 8). **b**, **c** The six significantly expressed genes detected by PCR validation in GF mice were verified in the hippocampus of *E. coli* LPS-treated mice (LPS, *n* = 8; control, *n* = 8) **b** and of “depression microbiota” recipient mice (depression, *n* = 8; Healthy, *n* = 8) **c**. Data represent the mean ± SEM. * (*p* < 0.05, FDR < 0.1), ** (*p* < 0.01, FDR < 0.1)

**Fig. 3:**
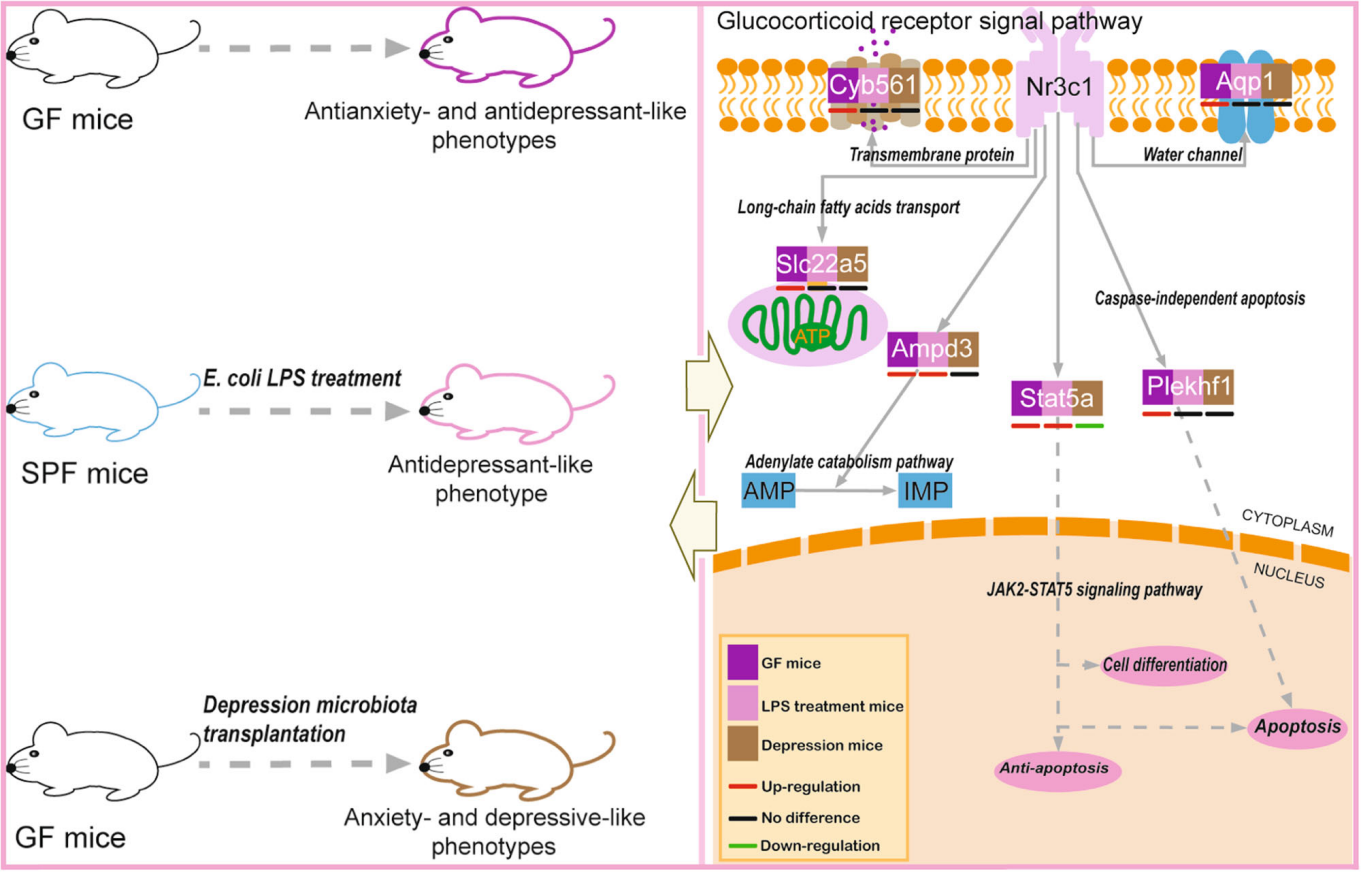
Brief summary of the entire research. The left panel shows the behavioral phenotypes for the three mouse models. The right panel illustrates the expression and biological functions of the six glucocorticoid receptor pathway genes. Abbreviations: AMP, adenosine monophosphate; IMP, inosine monophosphate; Nr3c1, glucocorticoid receptor

We used PCR to validate the 23 differentially expressed genes in microarray analysis. Six genes, including *Slc22a5*, *Aqp1*, *Stat5a*, *Ampd3, Plekhf1* and *Cyb561* were upregulated in GF mice compared with SPF mice (Fig. 2a). Moreover, these six significantly expressed genes were further validated in the hippocampus of *E. coli* LPS-treated mice and “depression microbiota” recipient mice. Two of them, *Stat5a* and *Ampd3* were upregulated in *E. coli* LPS-treated mice (*p* value < 0.05, FDR < 0.1) (Fig. 2b). However, *Stat5a* (FC = 0.84) was downregulated in “depression microbiota” recipient mice compared with “healthy microbiota” recipient mice (*p* value < 0.01, FDR < 0.1) (Fig. 2c). The details of these genes in three mouse models are displayed in Table 2 and Fig. 3.

**Table 2:** Details of the six significant genes identified in the three mouse models

	GF mice	LPS treatment mice	Depression mice
	Antianxiety- and antidepressant-like phenotypes	Antidepressant-like phenotype	Anxiety- and depressive-like phenotypes
*Slc22a5*	↑	—	—
*Aqp1*	↑	—	—
*Stat5a*	↑	↑	↓
*Ampd3*	↑	↑	—
*Plekhf1*	↑	—	—
*Cyb561*	↑	—	—

### *E. coli* LPS-treated mice show decreased basal serum cortisol levels and the other two mouse models show no change

**Fig. 4:**
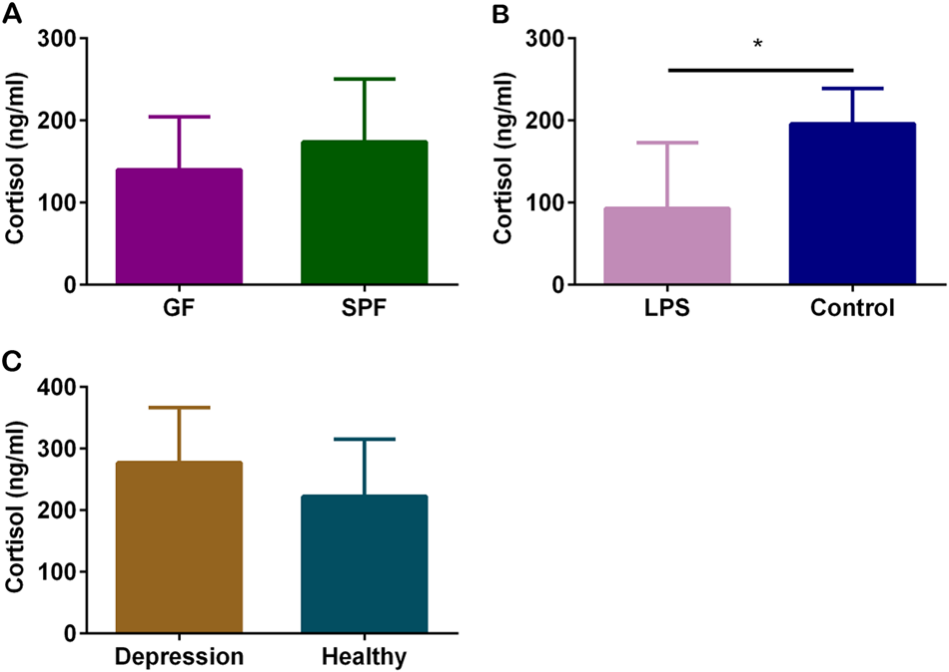
The basal serum cortisol levels in three mouse models. **a** The basal serum cortisol levels were quantified between germ-free (GF) mice (*n* = 6) and specific pathogen-free (SPF) mice (*n* = 6), **b** between *E. coli* lipopolysaccharide (LPS)-treated mice (*n* = 6) and control mice (*n* = 6) and **c** between “depression microbiota” recipient mice and “healthy microbiota” recipient mice (Depression, *n* = 6; Healthy, *n* = 6). Data represent the mean ± SEM. * *p* < 0.05

In present study, *E. coli* LPS-treated mice showed a decreased basal serum cortisol levels compared with control mice (Fig. 4b, *p* < 0.05). However, the basal serum cortisol levels did not change between GF mice and SPF mice (Fig. 4a), as well as between “depression microbiota” recipient mice and “healthy microbiota” recipient mice (Fig. 4c).

## Discussion

Recently, studies on the effects of gut microbiota on mice behaviors have been highlighted, and absence of microbiota may lead to behavioral abnormalities in mice through the HPA axis^20,21^. In current study, GF mice showed antianxiety- and antidepressant-like behaviors, and the targeted microarray analysis found 23 upregulated glucocorticoid receptor pathway genes in the hippocampus of GF mice. These genes are highly associated with neurological disorders. PCR validation confirmed that six genes (*Slc22a5*, *Aqp1*, *Stat5a*, *Ampd3, Plekhf1*, and *Cyb561*) were upregulated in the hippocampus of GF mice compared with SPF mice. In addition, *E. coli* LPS-treated mice showed antidepressant-like behavior, but did not show antianxiety-like behavior compared with the control mice. Of these six genes, two (*Stat5a* and *Ampd3*) were upregulated in *E. coli*-derived LPS-treated mice. Moreover, the “depression microbiota” recipient mice exhibited anxiety- and depressive-like behaviors^14^, and the *Stat5a* gene was downregulated in their hippocampus compared with the “healthy microbiota” recipient mice. In addition, basal serum cortisol levels reduced in *E. coli*-derived LPS-treated mice model, but did not change in GF mice model and “depression microbiota” recipient mice model.

Consistent with our previous research^14,18^, GF mice exhibited increased percentage of center distance and decreased immobility time compared with SPF mice, suggesting that absence of gut microbiota can lead to antianxiety- and antidepressant-like behaviors. Furthermore, Neufeld et al. have suggested that Swiss Webster female GF mice exhibited antianxiety-like behavior^21^. However, Crumeyrolle-Arias et al. have demonstrated that F344 GF rats displayed anxiety-like behavior compared with SPF rats^22^. We speculated that the different behavioral phenotypes in mice and rats may be related to the genetic background differences^27^.

Plasma ACTH and/or corticosterone levels in GF mice were higher after acute stress than SPF mice, but the basal cortisol levels showed no difference^19,20^. Our current study also showed there was no difference in basal serum cortisol levels between GF mice and SPF mice. However, K. M. Neufeld et al. showed that plasma corticosterone levels in GF mice were increased compared with SPF mice, but the authors also speculated that the elevated plasma corticosterone may be due to the stress of acclimatization^21^. These studies suggested that gut microbiota can affect the reactivity of the HPA axis. Furthermore, abnormal glucocorticoid levels can induce behavioral changes^28,29^. In our research, six glucocorticoid receptor pathway genes (*Slc22a5*, *Aqp1*, *Stat5a*, *Ampd3, Plekhf1*, and *Cyb561*) were upregulated in GF mice, but *Stat5a* was downregulated in “depression microbiota” recipient mice. Hence, we inferred that *Stat5a* may be the key mediator in the effects of glucocorticoid on the depressive-like behavior of mice. In addition, both GF mice and *E. coli* LPS-treated mice showed antidepressant-like behavior, and two glucocorticoid receptor pathway-related genes (*Stat5a* and *Ampd3*) were upregulated in the two models. We inferred that these two genes may be related to antidepressant-like behavior. Taken together, these findings may provide a further insight into the pathogenesis of depression, and suggest that the *Stat5a* gene is an important gene in depression.

The janus tyrosine kinase-signal transducer and activator of transcription (STAT) pathway is a pathway by which cells respond to cell growth as well as a variety of extracellular stimuli^30,31^. Stat5a is a member of the STAT family, which mediates intracellular signaling of the cytokine cell surface receptors, and transmits it to the nucleus^31,32^. It is also essential for cell differentiation, cell proliferation, cell apoptosis^32^, cell cycle regulation, and anti-apoptosis^31^. Sun et al. have found the *Stat5a* gene was increased in the hippocampus of rats after focal cerebral ischemia and reperfusion, indicating that *Stat5a* may play a protective role in damaged nerve cells^31^. However, the JAK2-STAT5 signaling pathway mediates interleukin-3-induced activation of microglia, which is associated with the pathogenesis of multiple sclerosis, Alzheimer’s disease (AD) and Parkinson’s disease^33^. Moreover, locomotor activity, feeding behavior, aggressive and exploratory behaviors may also be associated with cytokine-activated STAT5 signaling^34^. Growth hormone treatment can improve cognitive function^35^, which was accompanied with activation of STAT5 signaling pathway in mouse brain^34^. Hence, we speculated that the *Stat5a* may be associated with depression, and downregulation can lead to depressive-like behavior.

Aquaporins (AQPs) are the water channels that can transport water across cell membrane in many tissues^36^. Upregulation of Aquaporin 1 (AQP1) caused hippocampal cell edema and delayed cell death following traumatic brain injury^37^, showing that sometimes it protects against neuronal cell damage^38^. However, AQP1 protein overexpression was associated with vacuolization in the astroglial cytoplasm in CA1 astrocytes, which may cause cell apoptosis^39^. AQP1 may also lead to cytotoxic edema of the hippocampus by increasing the intracellular pH^40^. Furthermore, AQP1 has been reported to be abnormal expression in the brain of AD patients^41^. These studies suggest that the *Aqp1* gene may have protective or harmful effects on nerve cells.

*Plekhf1* is considered to initiate caspase-independent apoptosis through the lysosomal-mitochondrial pathway^42^. But to our knowledge, there is little study on its role in nervous system. We speculated it may affect the nervous system through cell apoptosis. Cytochrome b561 is considered to be the major transmembrane protein in catecholamines and neuropeptide-secreting vesicles of the pituitary and other neuroendocrine tissues^43^. It can transport electrons across the lipid bilayer to provide transmembrane reduction equivalents, to support dopamine β-hydroxylase activity and peptidyl α-amidating monooxygenase activity^44,45^. Dopamine β-hydroxylase can hydroxylate dopamine to produce norepinephrine^46^. Dopamine and norepinephrine as neurotransmitters can directly affect behavior.

The *Slc22a5* gene encodes an organic cation/carnitine transporter that plays an important role in the pathway of long-chain fatty acids through the internal mitochondrial membrane for β-oxidation to produce ATP^47^. Carnitine and/or acetylcarnitine supplementation benefits AD and depression, as it not only produces ATP by mitochondrial oxidation, but it may enhance mitochondrial function and provide antioxidant effects by providing acetyl moieties^48^. Studies have shown that *Slc22a5* expressed in vascular endothelial cells can transport acetylcarnitine through the blood–brain barrier, regulating the concentration of carnitine and acetyl moieties on both sides of the blood–brain barrier^47^. In our research, the elevated *Slc22a5* in the hippocampus of GF mice suggests that carnitine transport and/or energy production may be increased, and thus may play a protective role. Furthermore, adenosine monophosphate deaminase 3 (Ampd3) is a key enzyme in the catabolic pathway of adenylate that breaks adenosine monophosphate into inosine monophosphate, and eventually produces uric acid^49,50^. Ampd3 is ubiquitously expressed; it can regulate the energy metabolism of the body and may regulate the energy balance^50^. ATP mediates lipid and protein synthesis, buffers intracellular calcium, and regulates apoptosis and resilience pathways^51^. Energy metabolic disorder is strongly associated with depression^52–54^. Therefore, ATP plays an important role in the brain. In present study, *Slc22a5* and *Ampd3* were upregulated in GF mice; hence we speculated they may affect brain by modulating ATP levels in hippocampus.

Previous study have shown abnormal gut microbiota can lead to reduced integrity of the intestinal barrier, leading to increased leakage of LPS, which can activate systemic inflammation^55^. Prevention of intestinal barrier impairment and decreasing circulating LPS levels by a probiotic treatment can attenuate the HPA axis response to stress^56^. In current study, oral *E. coli* LPS treatment induced abnormal behavior and increased glucocorticoid receptor pathway genes in mice. These results were consistent with previous findings that *E. coli* colonization in GF mice enhanced the HPA axis response to stress^20^. Moreover, basal serum cortisol levels were decreased in *E. coli* LPS-treated mice in current studies, we speculated that the feedback regulation of HPA axis might be involved.

*E. coli* LPS in gut has an immune stimulation function, and can establish immune tolerance in infants and reduce the incidence of autoimmune diabetes^57^. GF mice without gut-derived LPS may lack this immune tolerance. Furthermore, different gut bacteria-derived LPS can affect each other’s immune stimulating functions^57^, and we speculated that the “depression microbiota” recipient mice, which exhibited increased relative abundances of *Actinobacteria* and decreased *Bacteroidetes* in gut^14^ may also have abnormal immune tolerance. In addition, the abnormal immune system can lead to abnormal behaviors and elevated serum corticosterone baseline levels^58^. Hence, we speculated that gut microbiota may affect the HPA axis reactivity by regulating immune tolerance.

There are several limitations in our study. First, we did not conduct gene overexpression or knockdown tests, to further verify that key glucocorticoid receptor pathway gene influences behaviors, thus, we will further verify the present results in future studies. Second, we did not perform proteomics analysis to explore the exact protein changes caused by the upregulated and downregulated genes. We will further validate our results by combining proteomics and metabolomics. Third, oral LPS can induce abnormal behavior and gene expression in current study, but the mechanism is not clear. We speculated it may be associated with abnormal immune system. In next step, we will further explore the mechanism of oral LPS treatment affecting the mouse behavior and gene expression. Fourth, other pathways may also affect the glucocorticoid receptor pathway genes, so further studies are required to clarify whether other pathways are involved in the expression of glucocorticoid receptor pathway genes.

In summary, absence of gut microbiota induced antianxiety- and antidepressant-like phenotypes in GF mice, and *E. coli* LPS-treated mice exhibited antidepressant-like behavior, whereas the “depression microbiota” recipient mice showed anxiety- and depressive-like behaviors^14^, suggesting the gut microbiota affects depressive- and anxiety-like behaviors. Furthermore, glucocorticoid receptor pathway genes were upregulated in the hippocampus of mice with antianxiety- and/or antidepressant-like phenotypes. However, they were downregulated in mice with anxiety- and depressive-like phenotypes. These results indicated that the gut microbiota may lead to behavioral abnormalities in mice through the glucocorticoid receptor pathway. In present study, we provided new insight into the molecular mechanisms by which gut microbiota influence depressive-like behavior and proposed a new treatment target against depression.

## Electronic supplementary material

Supplementary figure legends

Supplementary figure S1

Supplementary figure S2

Supplementary figure S3

Supplementary method legends

Supplemental methods S1

Supplementary table legends

Supplementary Table. S1

Supplementary Table. S2
