# Supplementary material for: Gut microbiota regulates mouse behaviors through glucocorticoid receptor pathway genes in the hippocampus
Source: Transl Psychiatry. 2018 Sep 7;8:187. doi: 10.1038/s41398-018-0240-5 (PMC6128920; doi:10.1038/s41398-018-0240-5)
Supplement: Supplementary file 1 — Supplementary figure legends [file 41398_2018_240_MOESM1_ESM.docx]

**Supplementary figure legends**

**Supplementary** **Fig. S****1.** Twenty-three differentially genes were highly related to neurological disease. Ingenuity Pathway Analysis software was used to analyze the enrichment of 23 differentially expressed genes and diseases/functions. *P*-value was expressed as negative logarithm (-log).

**Supplementary Fig. S****2.** Molecular and cellular functions of the 23 differentially expressed genes in microarray analysis results. Ingenuity Pathway Analysis showed these genes were highly related with cell development, cell proliferation, cell death and cell survival. *P*-value was expressed as negative logarithm (-log).

**Supplementary Fig. S3.** The relevant networks of 23 significantly differentially expressed genes in microarray analysis results.
