# Supplementary figures and images for: Gut microbiota regulates mouse behaviors through glucocorticoid receptor pathway genes in the hippocampus
Source: Transl Psychiatry. 2018 Sep 7;8:187. doi: 10.1038/s41398-018-0240-5 (PMC6128920; doi:10.1038/s41398-018-0240-5)

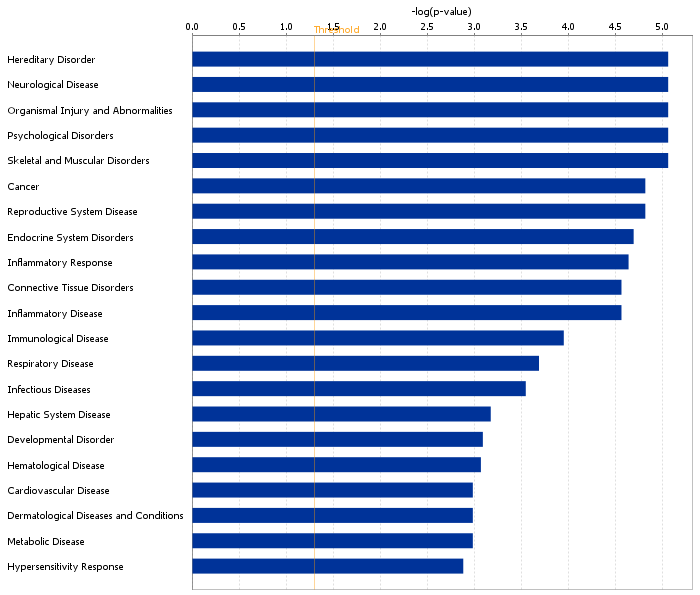

Supplement: Supplementary file 2 — Supplementary figure S1 [file 41398_2018_240_MOESM2_ESM.png]

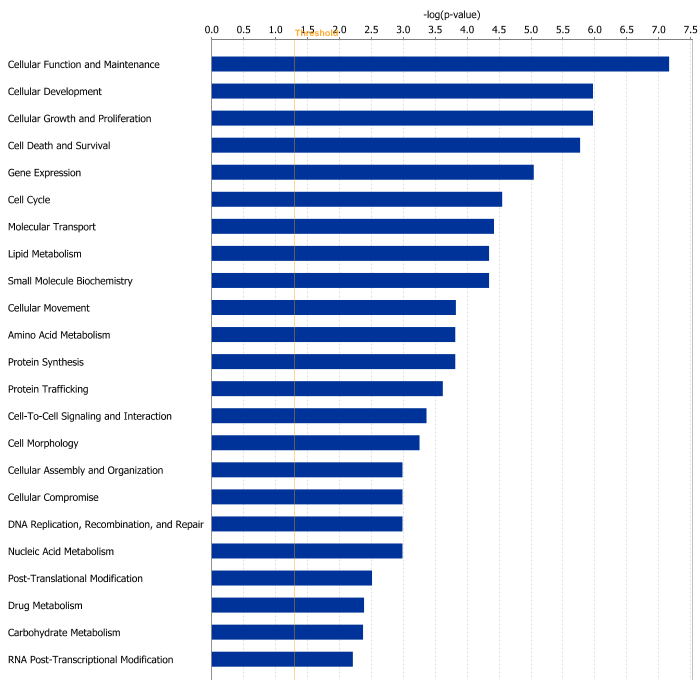

Supplement: Supplementary file 3 — Supplementary figure S2 [file 41398_2018_240_MOESM3_ESM.tif]

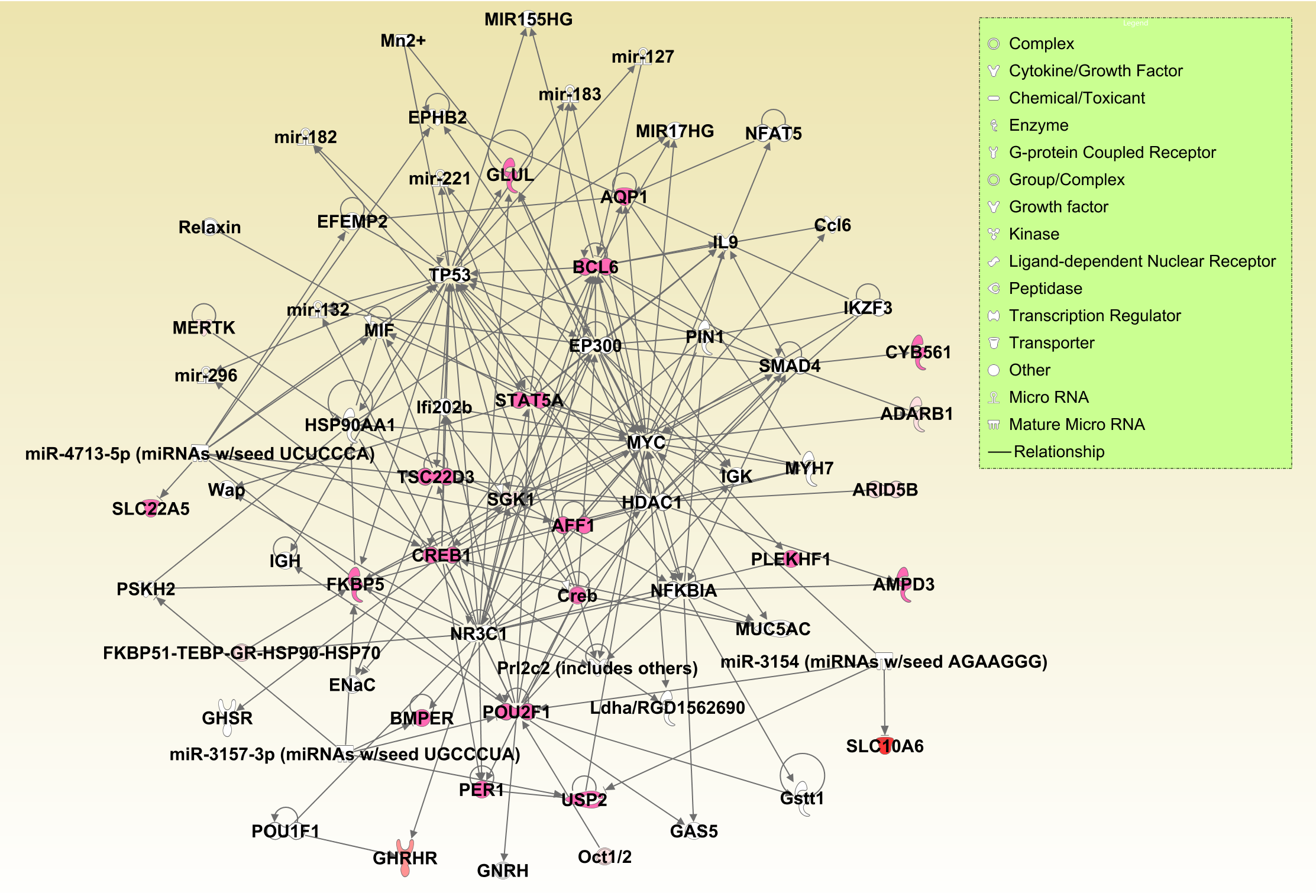

Supplement: Supplementary file 4 — Supplementary figure S3 [file 41398_2018_240_MOESM4_ESM.tif]

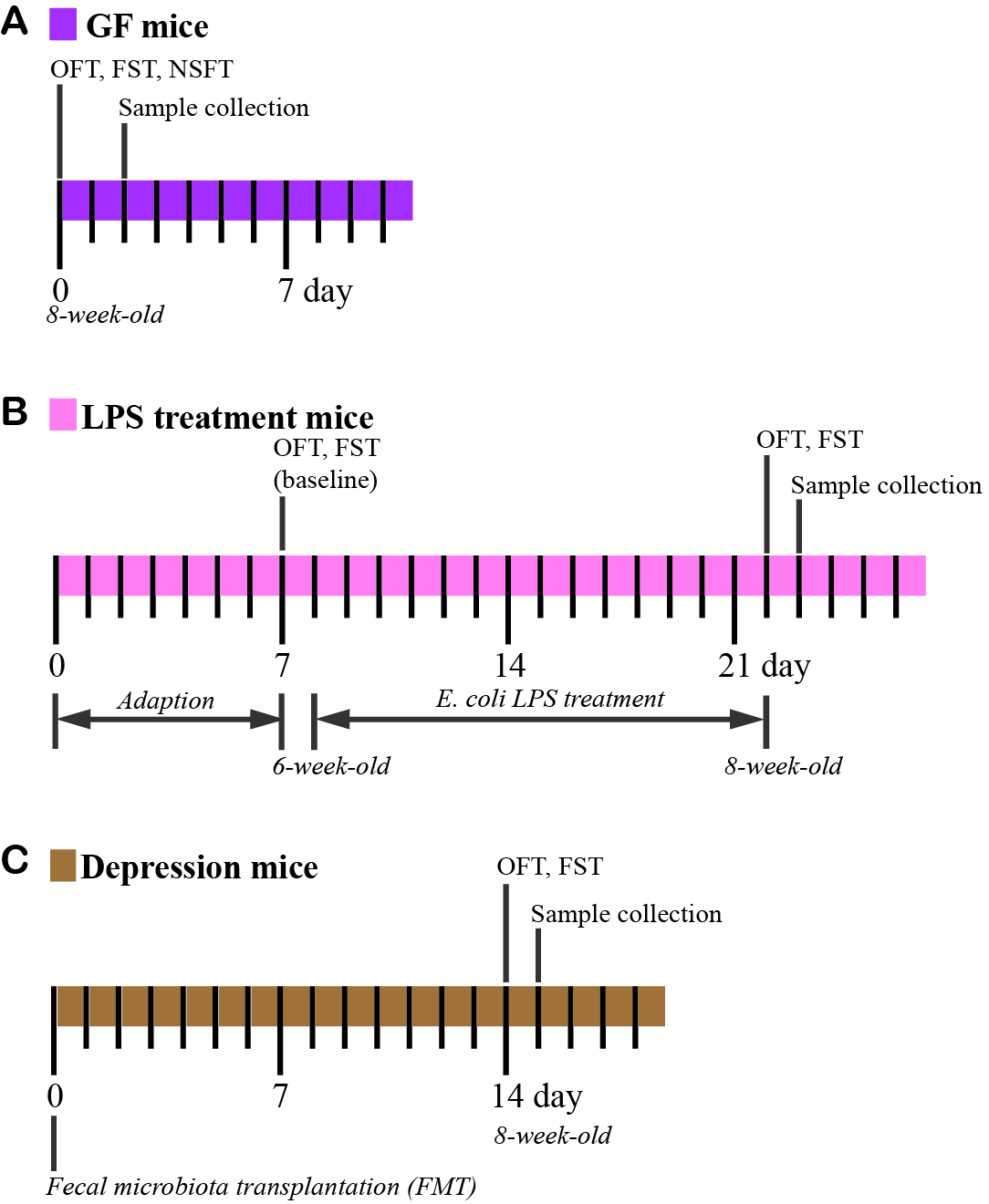

Supplement: Supplementary file 6 — Supplemental methods S1 [file 41398_2018_240_MOESM6_ESM.tif]
