# Supplementary material for: Gut microbiota regulates mouse behaviors through glucocorticoid receptor pathway genes in the hippocampus
Source: Transl Psychiatry. 2018 Sep 7;8:187. doi: 10.1038/s41398-018-0240-5 (PMC6128920; doi:10.1038/s41398-018-0240-5)
Supplement: Supplementary file 5 — Supplementary method legends [file 41398_2018_240_MOESM5_ESM.docx]

**Supplementary method legends**

**Supplementary method. S1** The timelines for each set of experiments.

(A) The open field test (OFT), the forced swim test (FST) and the novelty suppressed feeding test (NSFT) were performed when the germ-free (GF) (n = 19) and specific pathogen free (SPF) (n = 20) mice were eight weeks old. (B) The OFT and the FST of *E. coli* LPS-treated mice (n = 13) and control mice (n = 13) were performed at the age of six weeks and eight weeks respectively. (C) The fecal microbiota transplantation (FMT) was performed at the age of six weeks. The OFT and the FST of the depression microbiota recipient mice (n = 30) and healthy microbiota recipient mice (n = 30) were performed at the eight weeks old.
