# Supplementary material for: Gut microbiota regulates mouse behaviors through glucocorticoid receptor pathway genes in the hippocampus
Source: Transl Psychiatry. 2018 Sep 7;8:187. doi: 10.1038/s41398-018-0240-5 (PMC6128920; doi:10.1038/s41398-018-0240-5)
Supplement: Supplementary file 7 — Supplementary table legends [file 41398_2018_240_MOESM7_ESM.docx]

**Supplementary Table legends**

**Supplementary Table. S1** Microarray gene table.

**Supplementary Table. S2** The primer sequences
