# Supplementary material for: Gut microbiota regulates mouse behaviors through glucocorticoid receptor pathway genes in the hippocampus
Source: Transl Psychiatry. 2018 Sep 7;8:187. doi: 10.1038/s41398-018-0240-5 (PMC6128920; doi:10.1038/s41398-018-0240-5)
Supplement: Supplementary file 8 — Supplementary Table. S1 [file 41398_2018_240_MOESM8_ESM.docx]

**Supplementary Table. S1.** Microarray gene table

| Loction | Symbol | Description | Loction | Symbol | Description |
| --- | --- | --- | --- | --- | --- |
| A01 | Adarb1 | Adenosine deaminase, RNA-specific, B1 | **E01** | Pdcd7 | Programmed cell death 7 |
| A02 | Aff1 | AF4/FMR2 family, member 1 | **E02** | Pdgfrb | Platelet derived growth factor receptor, beta polypeptide |
| A03 | Ak2 | Adenylate kinase 2 | **E03** | Pdp1 | Pyruvate dehyrogenase phosphatase catalytic subunit 1 |
| A04 | Ampd3 | Adenosine monophosphate deaminase 3 | **E04** | Per1 | Period homolog 1 (Drosophila) |
| A05 | Angptl4 | Angiopoietin-like 4 | **E05** | Per2 | Period homolog 2 (Drosophila) |
| A06 | Anxa4 | Annexin A4 | **E06** | Pik3r1 | Phosphatidylinositol 3-kinase, regulatory subunit, polypeptide 1 (p85 alpha) |
| A07 | Aqp1 | Aquaporin 1 | **E07** | Pld1 | Phospholipase D1 |
| A08 | Arid5b | AT rich interactive domain 5B (MRF1-like) | **E08** | Plekhf1 | Pleckstrin homology domain containing, family F (with FYVE domain) member 1 |
| A09 | Asph | Aspartate-beta-hydroxylase | **E09** | Pou2f1 | POU domain, class 2, transcription factor 1 |
| A10 | Atf4 | Activating transcription factor 4 | **E10** | Pou2f2 | POU domain, class 2, transcription factor 2 |
| A11 | Bcl6 | B-cell leukemia/lymphoma 6 | **E11** | Rasa3 | RAS p21 protein activator 3 |
| A12 | Bmper | BMP-binding endothelial regulator | **E12** | Rgs2 | Regulator of G-protein signaling 2 |
| B01 | Calcr | Calcitonin receptor | **F01** | Rhob | Ras homolog gene family, member B |
| B02 | Cebpa | CCAAT/enhancer binding protein (C/EBP), alpha | **F02** | Rhoj | Ras homolog gene family, member J |
| B03 | Cebpb | CCAAT/enhancer binding protein (C/EBP), beta | **F03** | Sesn1 | Sestrin 1 |
| B04 | Col4a2 | Collagen, type IV, alpha 2 | **F04** | Sgk1 | Serum/glucocorticoid regulated kinase 1 |
| B05 | Creb1 | CAMP responsive element binding protein 1 | **F05** | Slc10a6 | Solute carrier family 10 (sodium/bile acid cotransporter family), member 6 |
| B06 | Creb3 | CAMP responsive element binding protein 3 | **F06** | Slc19a2 | Solute carrier family 19 (thiamine transporter), member 2 |
| B07 | Creb3l4 | CAMP responsive element binding protein 3-like 4 | **F07** | Slc22a5 | Solute carrier family 22 (organic cation transporter), member 5 |
| B08 | Ctgf | Connective tissue growth factor | **F08** | Snta1 | Syntrophin, acidic 1 |
| B09 | Cyb561 | Cytochrome b-561 | **F09** | Sphk1 | Sphingosine kinase 1 |
| B10 | Ddit4 | DNA-damage-inducible transcript 4 | **F10** | Spsb1 | SplA/ryanodine receptor domain and SOCS box containing 1 |
| B11 | Diras2 | DIRAS family, GTP-binding RAS-like 2 | **F11** | Stat5a | Signal transducer and activator of transcription 5A |
| B12 | Dusp1 | Dual specificity phosphatase 1 | **F12** | Stat5b | Signal transducer and activator of transcription 5B |
| C01 | Edn1 | Endothelin 1 | **G01** | Tbl1xr1 | Transducin (beta)-like 1X-linked receptor 1 |
| C02 | Ehd3 | EH-domain containing 3 | **G02** | Tnf | Tumor necrosis factor |
| C03 | Errfi1 | ERBB receptor feedback inhibitor 1 | **G03** | Tnfaip3 | Tumor necrosis factor, alpha-induced protein 3 |
| C04 | Fkbp5 | FK506 binding protein 5 | **G04** | Tsc22d3 | TSC22 domain family, member 3 |
| C05 | Fosl2 | Fos-like antigen 2 | **G05** | Usp2 | Ubiquitin specific peptidase 2 |
| C06 | Gdpd1 | Glycerophosphodiester phosphodiesterase domain containing 1 | **G06** | Usp54 | Ubiquitin specific peptidase 54 |
| C07 | Ghrhr | Growth hormone releasing hormone receptor | **G07** | Vdr | Vitamin D receptor |
| C08 | Glul | Glutamate-ammonia ligase (glutamine synthetase) | **G08** | Vldlr | Very low density lipoprotein receptor |
| C09 | Got1 | Glutamate oxaloacetate transaminase 1, soluble | **G09** | Xdh | Xanthine dehydrogenase |
| C10 | H6pd | Hexose-6-phosphate dehydrogenase (glucose 1-dehydrogenase) | **G10** | Zfp281 | Zinc finger protein 281 |
| C11 | Has2 | Hyaluronan synthase 2 | **G11** | Zfp36 | Zinc finger protein 36 |
| C12 | Hnrpll | Heterogeneous nuclear ribonucleoprotein L-like | **G12** | Zhx3 | Zinc fingers and homeoboxes 3 |
| D01 | Il10 | Interleukin 10 | **H01** | Actb | Actin, beta |
| D02 | Il1rn | Interleukin 1 receptor antagonist | **H02** | B2m | Beta-2 microglobulin |
| D03 | Il6 | Interleukin 6 | **H03** | Gapdh | Glyceraldehyde-3-phosphate dehydrogenase |
| D04 | Il6ra | Interleukin 6 receptor, alpha | **H04** | Gusb | Glucuronidase, beta |
| D05 | Klf13 | Kruppel-like factor 13 | **H05** | Hsp90ab1 | Heat shock protein 90 alpha (cytosolic), class B member 1 |
| D06 | Klf9 | Kruppel-like factor 9 | **H06** | MGDC | Mouse Genomic DNA Contamination |
| D07 | Lox | Lysyl oxidase | **H07** | RTC | Reverse Transcription Control |
| D08 | Mertk | C-mer proto-oncogene tyrosine kinase | **H08** | RTC | Reverse Transcription Control |
| D09 | Mt1 | Metallothionein 1 | **H09** | RTC | Reverse Transcription Control |
| D10 | Mt2 | Metallothionein 2 | **H10** | PPC | Positive PCR Control |
| D11 | Nfkbia | Nuclear factor of kappa light polypeptide gene enhancer in B-cells inhibitor, alpha | **H11** | PPC | Positive PCR Control |
| D12 | Nr3c1 | Nuclear receptor subfamily 3, group C, member 1 | **H12** | PPC | Positive PCR Control |
